# Barriers and facilitators to addressing sedentary behaviour and physical inactivity among nursing home residents: a qualitative study

**DOI:** 10.1186/s12877-025-06272-2

**Published:** 2025-08-20

**Authors:** Yihan Mo, Helen Yue-lai Chan, Yuxin Zhou, Linghui Chen, Guanxiu Tang, Anna Bone, Matthew Maddocks, Catherine Evans

**Affiliations:** 1 Cicely Saunders Institute of Palliative Care, Policy and Rehabilitation, King’s College London, London, UK; 2 The Nethersole School of Nursing, Faculty of Medicine, The Chinese University of Hong Kong, Hong Kong, SAR China; 3 Department of Nursing, Zhongda Hospital Southeast University, Nanjing, China; 4 Department of Geriatrics, The Third Xiangya Hospital, Central South University, Changsha, China

**Keywords:** Interviews, Nursing homes, Physical inactivity, Qualitative, Sarcopenia, Sedentary behaviour

## Abstract

**Background:**

Sedentary behaviour and physical inactivity contribute to muscle-related decline (e.g., sarcopenia), chronic disease and disability. This study aims to (1) explore nursing home residents’ and staff’s perspectives and experiences regarding interventions addressing sedentary behaviour and physical inactivity; (2) refine a Theory of Change logic model for such interventions in nursing homes.

**Methods:**

An exploratory qualitative study design. In-person interviews with 14 residents and 12 staff members at two urban nursing homes in China. Data were analysed applying codebook thematic analysis, mapped onto the Ecological Social Theory encompassing individual (micro), organisational (meso), family (exo) and societal (macro) contexts.

**Results:**

Four themes were identified. (1) Microsystem: individual physical, psychosocial, cognitive, and educational constraints hindered participation in physical activity, while perceived potential for benefit encouraged participation. (2) Mesosystem: social interaction between residents impacted on activity participation both positively (e.g., encouragement) and negatively (e.g., peer pressure). Staff roles were key facilitators by coordinating intervention while insufficient activity management and resources constrained use. (3) Exosystem: family attitudes, support and bonds influenced the decision-making process and residents’ motivation in activities. (4) Macrosystem: sufficient funding was important to provide and sustain activity interventions in nursing homes. Societal ties with the community external to nursing homes boosted residents’ activity engagement.

**Conclusions:**

Multilevel factors from individual, organisational, family, and societal contexts were identified. A Theory of Change logic model was adapted by enhancing its applicability to specific context while maintaining its general framework. To optimise activity interventions in nursing homes, it is essential to enhance staff roles, refine activity management, leverage social interactions within nursing homes strategically, maintain family involvement, sustain financial input and collaborate with social organisations. Multilevel strategies are crucial for addressing the global challenges of sedentary behaviour and physical inactivity in nursing homes. Adapting the Theory of Change logic model to specific context opens opportunities for tailored application across diverse cultural settings.

**Clinical trial number:**

Not applicable.

**Supplementary Information:**

The online version contains supplementary material available at 10.1186/s12877-025-06272-2.

## Background

Sedentary behaviour and physical inactivity are prevalent among older adults in nursing homes [1]. Sedentary behaviour, referring to certain activities in a reclining, seated, or lying position requiring very low energy expenditure, is an independent predictor of metabolic risk [2]. It is distinct from physical inactivity, which is characterised by an insufficient amount of moderate-to-vigorous physical activity that fails to meet the recommended guidelines for a given age group [3]. Older adults in nursing homes spend on average 79% of their waking hours sedentary, with only 20% in low-intensity physical activities and 1% in moderate-to-vigorous activities [4]. Both sedentary behaviour and physical inactivity contribute to muscle-related decline (e.g., sarcopenia) [5], disability and higher mortality risk. The 2023 physical activity guideline for nursing home, asserts that minimising sedentary behaviour is equally important as increasing physical activity [6]. Among nursing home residents, replacing sedentary activities with low-intensity physical activity is likely more effective than moderate-to-vigorous physical activity to promote an active lifestyle and reduce mortality risk [7].

Nursing homes are defined as facilities with registered nurses who offer clinical support and oversight, and healthcare assistants who provide 24-hour functional support and care for people who require assistance with activities of daily life, and who often have complex health needs and increased vulnerability [8]. Numerous studies have explored factors influencing physical inactivity among nursing home residents. A qualitative study revealed three barriers to physical activity: inadequate support, pervasive institutional routines, and physical environment constraints [9]. A mixed-methods review examined environmental factors affecting physical activity among older residents across three levels: physical (accessible and safe living environments), social (supportive professionals and role of peers), and symbolic (policy and organisational values) [10]. A systematic review identified barriers (understaffing, fatigue, distrust) and facilitators (structured and tailored programmes, group exercise and frequent sessions) to implement physical activity programmes for individuals with dementia in nursing homes [11].

Although numerous studies have focused on the challenges of promoting physical activity in nursing homes, growing evidence points to interventions that can effectively support and enhance residents’ physical health. A systematic review [12] found that functionally based programmes such as walking and exercise training can effectively enhance physical health, even among residents with cognitive impairments. Similarly, a study [13] examined e-health and exergaming interventions (video gaming with an exercise component) and reported potential benefits in fall prevention and physical functioning, though they noted limited applicability to residents with severe impairments. For residents with dementia, tailored and nurse-led interventions have shown promise. A systematic review [14] summarised a variety of such interventions and emphasised their relevance and feasibility in dementia care, despite mixed evidence of effectiveness. Furthermore, another review [15] synthesised qualitative studies and emphasised that residents’ motivation and willingness to participate in physical activity are shaped by their attitudes, autonomy, and the extent to which programmes are personalised. These findings underscore the need to develop individualised, inclusive, and sustainable activity programmes that consider the diverse capacities and needs of nursing home residents.

While physical activity has been extensively explored, the combination of sedentary behaviour and physical inactivity among nursing home residents are less considered. Studies have either solely focused on factors impacting sedentary behaviour in nursing homes [16, 17], or explored older adults’ perspectives on both physical inactivity and sedentary behaviour, but only in the home setting [18]. The scarcity of studies on factors influencing sedentary behaviour and physical inactivity among nursing home residents, hampers efforts to address these issues. Understanding these factors is vital for developing targeted interventions to promote active lifestyles and improve overall well-being in this population.

This study aims to (1) explore perspectives and experiences of residents and staff in nursing homes to identify barriers and facilitators for addressing sedentary behaviour and physical inactivity; and (2) inform the refinement of a Theory of Change logic model describing context, components and outcomes for interventions addressing these issues in nursing homes within a specific cultural context.

## Methods

### Study design

This exploratory qualitative design study is part of a sequential multi-methods research project developing and testing the feasibility of a multicomponent tailored intervention targeting sedentary behaviour and physical inactivity among nursing home residents at risk of, or with sarcopenia. The qualitative results were reported following the COREQ checklist [19] to ensure comprehensive standards and transparency, while drawing on Braun and Clark’s guidelines for thematic analysis reporting [20].

### Setting

Two public-private partnership nursing homes with 85 beds and 120 beds, respectively located in cities in Hunan Province, China. The localities comprise high-aged populations, with in 2023, 22.2% of people aged 60 and older, above the national average of 21.1% [21]. The nursing homes were selected based on facility type (i.e., size, nursing home provision) and geographical location within Hunan province to reduce logistical challenges related to transportation, communication, and institutional policies.

### Sample size and selection of participants

Criterion-based purposive sampling was applied to residents (i.e., by age, risk of sarcopenia) to include participants central to the study's focus. Maximum variation purposive sampling was used for staff (i.e., by professional roles, work experiences) to reflect diverse perspectives on the study's topic. The planned sample size comprises approximately 20 residents and 10 staff, guided by the concept of information power [22] and related studies [23, 24], continuously evaluating participant group size based on study relevance and quality of data. Inclusion criteria for residents were those aged 60 years and over, with mental capacity to give informed consent, and at risk of sarcopenia. Residents’ risk of sarcopenia was identified using sarcopenia case-finding criteria recommended for research and clinical care using the Asian Working Group of Sarcopenia. The criteria consist of: (1) Presence of any of the following clinical conditions: functional decline or limitation; unintentional weight loss; depressive mood; cognitive impairment; repeated falls; malnutrition; and/or chronic conditions (e.g., heart failure, chronic obstructive pulmonary disease, diabetes mellitus, chronic kidney disease). And (2), if no clinical conditions above are present: low calf circumference (male < 34 cm, female < 33 cm), or score of Strength, Assistance in walking, Rising from a chair, Climbing stairs, and Falls (SARC-F) questionnaire ≥ 4, or score of SARC-F combined with Calf circumference (SARC-CalF) questionnaire ≥ 11. Inclusion criteria for staff were individuals working at the selected sites with three months or more experience in long-term care, including managers, registered nurses, and healthcare assistants.

Finally, 26 participants (14 residents and 12 staff members) included in this study. A total of 20 interviews were conducted, with 17 individual interviews (14 residents and 3 staff) and 3 small group interviews with 9 staff (Table 1). Although the final numbers slightly differed from the initial plan, the achieved sample was sufficient. No new themes emerged in the final interviews, and given the study's narrow focus, sample specificity, and high-quality data, the sample provided adequate information power to address the research aims. Of the 30 participants approached, 2 residents were unable to participate due to severe hearing impairment and lack of cognitive capacity, and 2 staff were excluded due to limited working experience. Most participants were female (residents 57% and staff 83%). Residents were mean age 78.7 years. Most residents had a primary education (*n* = 6), followed by junior high (*n* = 4) and high school (*n* = 3), with one resident being without formal education. Their duration of residence in nursing home was evenly distributed across short-term (≤ 1 year, *n* = 5), mid-term (1–3 years, *n* = 4), and long-term (≥ 3 years, *n* = 5). Professional roles of staff included managers, registered nurses, and healthcare assistants, with most (*n* = 7) having over three years of experience in nursing homes.

**Table 1:** Characteristics of participants (*n* = 26)

Characteristics of residents (*n* = 14)		Characteristics of staff (*n* = 12)	
Sex		Sex	
Female (n, %)	8 (57)	Female (n, %)	10 (83)
Age (years)		Age (years)	
Median (range)	79.5 (64–88)	Median (range)	34.0 (27–55)
Educational level		Professional role	
Withou formal education	1	Manager	3
Primary school (up to 6 years)	6	Registered nurse	5
Junior high school (up to 9 years)	4	Healthcare assistant	4
High school (up to 12 years)	3		
Duration of residence in nursing homes		Work experience in nursing homes	
≤ 1 year	5	≤ 1 year	4
1–3 years	4	1–3 years	1
≥ 3 years	5	≥ 3 years	7

### Recruitment

Residents and staff were separately identified, approached, and recruited face-to-face. For residents, study flyers were posted on bulletin boards in the nursing home sites. Researcher (YM) introduced the study to potential participants using an information sheet and, with staff assistance, gauged residents’ willingness to participate. Residents mental capacity to give informed consent was assessed according to the Mental Capacity Act Code of Practice, focusing on understanding, retaining and weighing up relevant information, and communicating their decision [25]. Residents were asked to talk through detail in the information sheet to check understanding and recall, encouraged to discuss the pros and cons of participating with staff and/or family, and to express their choice clearly verbally or through other means [25]. Researcher (YM) made every effort to maximise residents’ autonomy, for example for those with impaired capacity, iterative communication methods (i.e., oral, and written information, body language) were used [26]. Any questions were addressed, and at least 24 h were provided for decision-making.

For staff, the study was introduced during informal meetings. They were given at least 24 h to decide on participation, and informed that their decision would not affect their work or rights. Separate individual or small group interviews were scheduled for managers, nurses, and healthcare assistants, following informed consent.

Participation in the study was entirely voluntary, and no financial or material incentives were offered to either residents or staff. Participants were informed that they would not receive any personal or financial benefit from taking part, and that their decision to participate or not would have no impact on their care, treatment, or employment status. Participants were assured that they could withdraw from the study at any time and for any reason, without any adverse impact on their accommodation or care (for residents), or their employment or working conditions (for staff).

The researcher (YM) was highly attentive to the potential vulnerability of nursing home residents due to advanced age and multimorbidity. All participants were also informed about the potential consequences of participation, including the minimal risks and possible fatigue and emotional discomfort during interviews.

### Data collection

Data collection involved semi-structured interviews conducted between January and March 2023, carried out individually with residents and either individually or in small groups with staff members. All data collection was completed by YM, a local of the study area, fluent in the local dialect and customs, and a registered nurse and PhD candidate in nursing trained in qualitative research and supported by an experienced qualitative researcher (CE). Interviews were conducted face-to-face in Chinese and with the researcher’s fluency in the local dialect enabling rapport with participants and understanding of cultural cues and dynamics during interviews. Individual interviews with residents were conducted in their rooms or public areas based on their choices. The median interview length was 49 min (range: 23–106 min), with adjustment into shorter interviews according to the situation of the interviewee. Individual or small group interviews with staff were undertaken in the nursing home, in an office or meeting area. In small group interview, semi-structured interviews are conducted with several people at the same time. It sought to prioritise individual input within the group, with for example directing questions to each person to ensure each could contribute [27].

To minimise fatigue and distress during interviews, participants were offered the option of multiple shorter interview sessions, with breaks as needed. For example, one resident completed the interview in three sessions averaging 15 min each. During interviews, participants were regularly reminded that they could pause or stop the interview at any time. The interviewer monitored participants for signs of discomfort and responded by offering breaks or stopping the interview if requested. No participant reported distress or chose to withdraw during or after the interview.

The interview topic guides included sections for collecting demographic data, exploring participants’ experiences with sitting/lying/reclining time reduction and physical exercise, and identifying reasons for engaging in or avoiding sedentary reduction and exercise. The Behaviour Change Wheel, centred around Capability, Opportunity, Motivation-Behaviour model [28], informed the design of the interview topic guides. Questions were designed to assess residents’ physical abilities, residents’ and staff’s understanding, knowledge, and skills (Capability) related to reducing sedentary behaviour and increasing physical activity for nursing home residents (Behaviour). External factors that might facilitate or hinder physical activity, such as social support from staff or family, available resources, and the physical environment (Opportunity) were covered. Participants’ reflective (e.g., beliefs and intentions) and automatic (e.g., emotions and habits) responses (Motivation) were also considered. Interview topic guides were adjusted separately for residents, senior staff (managers and nurses), and healthcare assistants (Supplementary material). Pilot interview conducted by YM with a researcher (YZ) and a nursing home resident refined and confirmed the topic guide’s relevance and accessibility. Interviews were digitally audio-recorded with consent. Field notes recording interview process, contextual factors, participant responses and reflexive journaling recording personal reflections were completed after interviews and used in the data analysis.

### Data analysis

Interviews were transcribed verbatim by YM and a bilingual (Chinese and English) postgraduate student with transcription experience and checked for accuracy by YM. Data underwent codebook thematic analysis (framework method, primarily inductive with supplemental deductive) [29, 30]. This was achieved by combining inductive data analysis with deductive theoretical interpretation to enhance the relevance and applicability of results. MAXQDA 2020 software was used for analysis. The analysis process started with transcription and data familiarisation. Inductive coding was then conducted line by line by two Chinese researchers (YM and LC) in Chinese, for half of the interviews (*n* = 10). Four bilingual (Chinese and English) researchers (YM, HC, YZ and LC) reviewed the coding and translated the codes, sub-themes/themes, and key quotes in English. An English working analytical framework was developed after initial coding, reviewed, discussed, and agreed upon by the wider team (CE, MM and AB) and bilingual researchers (HC, YZ and LC), with iterations continuing until no new codes were identified. This framework was then applied to subsequent transcripts, with some codes combined and no new codes developed. Data were summarised into a framework matrix using a spreadsheet, included references to illustrative quotations.

The Ecological Social Theory [31] was used post-coding to contextualise and organise emerging themes and codes within a multi-system perspective, covering microsystem (individual), mesosystem (organizational), exosystem (family), and macrosystem (societal factors), that influence sedentary behaviour and physical inactivity in nursing homes. This theory recognises the ecology of human growth and development, emphasising the complex interplay of individual, socio-cultural, and environmental factors [31]. We interpreted and discussed the results with consideration of field notes and reflexive journaling. Our findings were reported in a descriptive way.

In addition, a Theory of Change logic model, previously developed through a systematic review [32], provided a theoretical model for interventions addressing sedentary behaviour and physical inactivity in nursing homes. Theory of Change is a pragmatic framework used to plan, describe and evaluate the processes through which a desired change or outcome is expected to occur [33]. Theory of Change presented in a logic model visually illustrate the relationships between intervention activities and desired outcomes, outlining underlying assumptions and contextual factors. The findings of this study were applied afterward to update and contextualise the model, particularly for Chinese nursing homes.

### Establishing rigour and trustworthiness

Credibility and auditability are crucial criteria for assessing the rigour and trustworthiness of qualitative data [34]. Three strategies were implemented to strengthen credibility and auditability: (a) audio-recording and verbatim transcription of the interviews, (b) investigator triangulation was applied during data analysis through independent coding followed by group meetings for data analysis and interpretation, (c) maintaining a detailed audit trail, which included comprehensive records of data collection methods, coding decisions, analysis procedures, and their rationales [34], and (d) data triangulation was achieved by including participants from diverse stakeholder groups, residents and staff in various roles (nurses, healthcare assistants, and managers), to ensure a broad range of perspectives on the phenomenon under study. Chinese transcripts were initially coded in their original language, then reviewed and translated by four bilingual team members, resulting in an English working analytical framework that was reviewed, discussed, and agreed upon by co-authors until no new codes emerged. In line with Braun and Clarke’s guideline on thematic analysis reporting [20], YM, as the lead researcher, used reflexive journaling to engage in critical reflections on how the position as a PhD student and registered nurse with limited clinical experience shaped interactions with participants and influenced the research process [35].

### Ethical considerations

Ethical approval for this study was obtained from the King’s College London (KCL) Research Ethics Committee (Ref: HR/DP-22/23-33808). The researcher (YM) conducted fieldwork in China with the support of the China Scholarship Council. Although not affiliated with a Chinese institution, formal permission to conduct the study was granted by the management of the two participating nursing homes in Hunan province, who acknowledged the KCL ethical approval as sufficient.

To protect participants’ privacy, all transcripts were anonymised by removing any personally identifiable information and assigning unique ID number to each participant. The audio recordings were securely stored on a password-protected device accessible only to the research team. After transcription, the audio files were deleted to further ensure confidentiality. The anonymised transcripts are stored securely and will be destroyed within the specified time in accordance with the ethically approved data management plan.

All procedures adhered to the principles of the Declaration of Helsinki [36], including obtaining written informed consent from all participants, ensuring autonomy, confidentiality, and the protection of vulnerable groups.

## Results

### Thematic findings

**Fig. 1:**
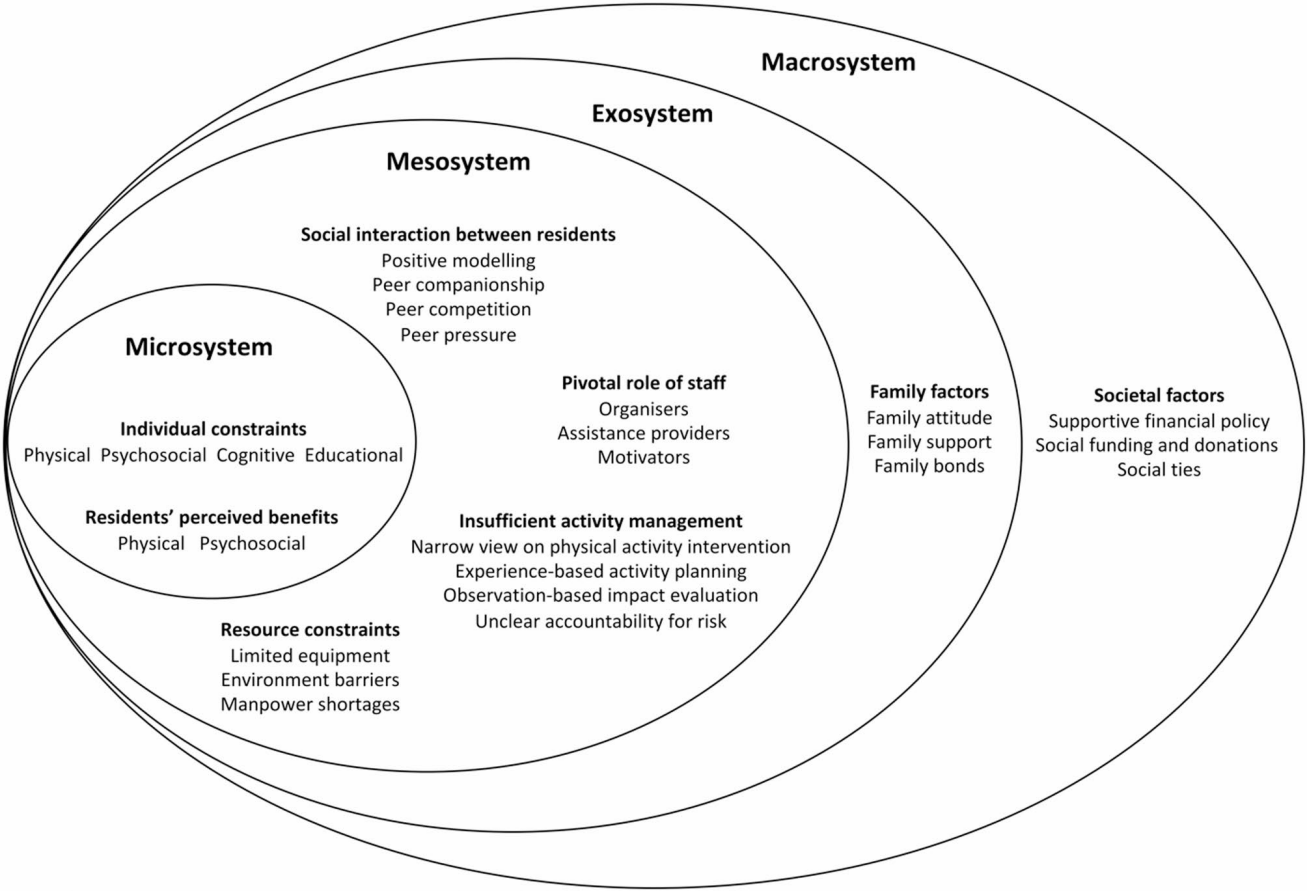
An Ecological Model [31] of barriers and facilitators for Interventions addressing sedentary behaviour and physical inactivity in nursing homes

The findings constructed an ecological system model of barriers and facilitators for interventions addressing sedentary behaviour and physical inactivity in nursing homes shown in Fig. 1. The barriers and facilitators encompassed four systems: (1) Microsystem: individual constraints and perceived advantages impact activity participation; (2) Mesosystem: organisational factors influence activity participation and intervention implementation; (3) Exosystem: Family factors impact initiation and continuation of activity participation; (4) Macrosystem: Societal factors enhance sustainability of activity intervention.

1. Microsystem: individual constraints and perceived benefits impact activity participation
  1. Physical, psychosocial, cognitive, and educational constraints

The significant challenges faced by residents in maintaining physical activity were their own physical constraints and capabilities. Many residents experienced a decline in physical capabilities (i.e., decreased mobility, lack of energy, feeling of tiring), which impeded both their willingness and ability to move freely. One resident described:

*“Many older adults don’t feel like moving when they get old. They lack energy*,* and their legs are not strong enough to walk…Take these older adults*,* for example*,* they don’t like to move*,* you know*,* some have difficulty on moving their hands and feet*,* some sit in a wheelchair*,* and if you ask them to do something*,* they take a long time to respond.” [Resident-N1F-04]*.

At the same time, residents’ acknowledgment of their limitations and concerns over not performing exercises correctly led to a negative self-image of appearing foolish in front of others, contributing to their hesitation, fearing mistakes and embarrassment when participating in activities. One resident expressed:

*“Sometimes I can’t perform certain movements well because I have only one hand that can move*,* and this hand can’t do it [patting her right hand with left hand]. I cannot do these exercises properly and they don’t look right either…I’m a bit afraid of making a fool of myself.” [Resident-N2F-10]*.

Residents’ fluctuating cognitive function was also described as a significant barrier to engagement in physical activities. Staff observed that residents with clear minds at times could participate well, but moments of cognitive confusion hindered their involvement:

*“Hmm*,* as I mentioned earlier*,* some of them will do it*,* but when their mind is not very clear*,* they definitely won’t do it*,* and they do the opposite. When their mind is clear*,* they do everything as asked. Um*,* it’s not a complete refusal to do it.” [Healthcare assistant-N2-02]*.

Lastly, residents with low levels of education were less confident, which negatively influences their engagement in new activities. One resident with three years of education expressed:

*“Actually*,* when I watch TV*,* I feel like my knowledge is not enough. I don’t understand the TV well*,* and whenever there’s a different activity*,* I don’t feel very confident.” [Resident-N2F-08]*.

2) Residents’ perceived physical and psychosocial benefits

Positive feedback from residents about perceived physical improvement from participating served as an effective facilitator and helped them reduce sedentary time and maintain physical activities. One resident described her experience:

*“I couldn’t move at all in the beginning*,* but now*,* after moving a bit every day and stretching*,* I feel much better. Look*,* now I don’t need anyone to help me go to the toilet…I stand up for a while after sitting for a long time…as it becomes uncomfortable. I have to adjust myself.” [Resident-N2F-08]*.

Similarly, residents perceived psychosocial benefits as key motivators for them engaging in physical activities. Some residents experience homesickness living in nursing homes. Engaging in physical activities not only enriched their daily lives but also helped to lift them out of feeling low in mood. One resident expressed this sentiment by sharing:

*“Oh*,* the time pass more quickly* [when doing physical activities], *right? Exercising the body makes the days pass quickly*,* and life becomes more comfortable. Otherwise*,* just sitting here*,* it’s not enjoyable. Another thing is homesickness. Sometimes*,* you think about how one child is doing*,* and then you think about how another child is doing. If you engage in physical activities*,* you forget about these things.” [Resident-N1F-04]*.

Physical activity is also seen to maintain dignity and respect as people age. A resident expressed his motivation of participating physical activity:

*“So*,* for the physical activity now*,* I do it for the sake of living a healthy and dignified life.” [Resident-N1M-03]*.

(2) Mesosystem: organisational factors influence activity participation and intervention implementation
  1. Social interaction between residents

Social interaction between residents plays a crucial role of shaping residents’ experience and participation in activities. Positive influencer, peer companionship and peer competition facilitate participation while peer pressure discourages residents’ involvement. In a group, a single enthusiastic participant can positively influence and motivate the rest of the group. A staff noted that:

*“In a group*,* there must be someone who empowers others. For example*,* we have someone*,* who is very proactive. No matter what activity*,* if you ask her*,* she will participate actively. Her enthusiasm*,* to some extent*,* can inspire other residents to join.” [Manager-N2-06]*.

Besides, engaging with others during activities can be a strong facilitator. This highlights the importance of positive social interaction and shared experiences in sustaining participation and enjoyment. One resident expressed that:

*“Ah*,* for activities*,* I want to be with others. I can learn from them. It’s not fun to play alone…Oh*,* I can’t persist on my own. I can’t keep it up alone.” [Resident-N2F-08]*.

In addition, a healthy dose of competition between peers was described as a motivator for residents to push their limits when participating physical activities. One resident explained that:

*“I think*,* you can see*,* looking at others*,* if they can do it*,* why can’t I? There’s a sense of encouragement*,* a motivating feeling. Feeling that if others can do it*,* I can too*,* because our situations are similar. We both have trouble moving one side. It gives a sense of motivation.” [Resident-N2F-10]*.

However, social dynamics can also introduce challenges. One resident described how peer pressure can threaten her confidence in activity participation and create feelings of exclusion:

*“If I’m in a wheelchair and you’re not*,* I won’t be able to compete with you*,* right? The person who isn’t in a wheelchair will be more capable*,* and they might think*,* ‘Oh*,* it’s not interesting to do this with her’.” [Resident-N1F-04]*.

2) The pivotal role of staff

Organisers, assistance providers and motivators are described as three key roles of staff during activity intervention. A stable and well-organised team of members who engage in regular communication and collaboration (activity coordinators), is favourable. One resident suggested that:

*“The exercise activities should be organised…not be random. I don’t want today someone comes to do it*,* tomorrow someone else…there should be a certain organisation*,* a certain structure. Relatively speaking*,* the personnel should be stable*,* they should communicate with each other*,* understand each other.” [Resident-N1M-03]*.

Besides, due to physical limitations of most of nursing home residents, assistance provided by staff ensures they can engage in physical activity effectively and safely. A staff described:

*“*[During activities] *Sometimes*,* they may not reach very high with their hands*,* so we can help them perform the movements more effectively……Currently*,* one older adult experiences difficulty using a walker*,* so it’s important that he is assisted by someone while engaging in short-distance walking exercises.” [Manager-N2-05].*

In addition, verbal encouragement and empowerment were emphasised in all staff interviews, to help enhance residents’ confidence and compliance to activities. One staff highlighted this by stating:

*“During these sessions*,* they receive light training while leveraging support*,* encouraging them to feel capable. This helps them to persist in exercises.” [Manager-N2-07]*.

3) Insufficient activity management

Several multifaceted challenges in activity management were explored, including a narrow view of physical activity, reliance on experience-based activity designing and observational impact evaluation, and unclear accountability for risks. Staff members exhibited a narrow view of physical activity and rehabilitation. They primarily perceived physical activity as appropriate only for individuals with fewer health issues. One staff member articulated this perspective, stating:

*“I think physical activity may be more suitable for those elderly people who have a higher level of participation and cooperation*,* and who are more active and have fewer underlying diseases. It seems that it may not be suitable for the elderly people here [with varying degrees of physical limitations and/or cognitive impairment].” [Nurse-N1-04]*.

Provision of physical activity were tailored according to the prolonged staff-resident relationship and understanding of residents’ conditions. It relied heavily on staff members’ personal knowledge and experiences, with little reference to guidelines:

*“Gradually*,* we are more acquainted with our residents. By our observations during the period of their living*,* we will then start exercising them based on our familiarity with them. Essentially*,* it’s about grading them and gradually training them…For now*,* we don’t follow any specific guideline*,* rely on our working experience.” [Manager-N2-05]*.

The impact of activities on residents was evaluated primarily through observation on visible improvements and subjective judgements, rather than regular systematic assessment using objective data. A staff member described this process:

*“After gradually exercising*,* you can see that he’s much better when he wakes up. He can walk*,* you know*,* with both feet*,* like a normal walk. Anyway*,* you can see it. We don’t go for regular assessments for each elderly person*,* or to say measure them regularly.” [Manager-N2-06]*.

Last, the issue of risk management and accountability posed a barrier to effective activity management. The ambiguity of assuming responsibility for potential risks related to physical activities limited the range and scope of activities offered to residents. One staff member explained:

*“… the third factor is also a very realistic one*,* which is the attitude of ‘better safe than sorry.’ For example*,* if I assist you in rehabilitation*,* there may be risks involved. Who should bear these risks? No one wants to take on the responsibility proactively. As a result*,* many rehabilitation activities or participation in activities might not proceed if there are risks involved and corresponding services have not been purchased.” [Nurse-N2-01]*.

4) Resource constraints

Resource constraints of limited equipment, environmental barriers of building design, and staff shortages were main challenges in facilitating regular physical activity for residents in nursing homes. The limited and outdated equipment available for use restricted the variety and effectiveness of physical activities that residents could engage in. One resident shared:

*“There is equipment in upper floor*,* but it’s very old and challenging to use. Some of the instruments seem to be broken.” [Resident-N1M-03]*.

Environmental barriers in nursing homes often posed challenges for residents, especially those with mobility issues. A resident described their experience with accessibility issues:

*“There’s a room with exercise equipment*,* and I can use it for exercise*,* stretching. However*,* there’s a slope in that place. I’m in a wheelchair*,* so I can’t go up by myself*,* I need someone to push me. When coming down*,* I don’t need help*,* but I’m a bit afraid because I can’t use brake well. I know there’s a brake*,* but I’m not sure if it works … Mm-hmm*,* anyway*,* I tried it a few times*,* but later*,* I stopped going.” [Resident-N2F-08]*.

Insufficient staffing and competing priorities of care were common barriers to providing physical activity and reducing sedentary time for residents. To meet all nursing home residents’ daily care needs in a timely manner, staff often take over tasks related to activities of daily living. Residents could complete some of these tasks with assistance, but staff opt to do them instead, as this approach is quicker than allowing residents to do it themselves. As a resident expressed:

*“I actually like doing a lot of things by myself*,* but I’m also a bit scared. So*,* I’ll say*,* ‘Let me stand and try it myself.’ Some of the kind staff will stand by and watch*,* letting me give it a try. But some will say*,* ‘What’s the point in trying? I can do it for you much faster.’ Sometimes*,* when they’re busy with many people*,* it’s hard for them to keep up*,* since they’re not just helping me—there are so many other older people waiting for their assistance too.” [Resident-N2F-10]*.

(3) Exosystem: Family factors impact initiation and continuation of activity participation

Family attitudes about physical activity for a resident influenced provision in the nursing homes. One staff member *[Manager-N2-07]* noted that obtaining approval from family or legal guardians was essential before initiating activities for nursing home residents. Family members with negative perceptions and attitudes to physical activity directly hindered provision, often leading to reluctance or avoidance in starting activities for older individuals. A staff member explained:

*“Some families believe that because the older adults are advanced in age*,* there’s no need to trouble them with activities. They think it’s better to let them peacefully live out their lives*,* taking care of their basic needs such as food*,* clothing*,* shelter*,* and hygiene*,* ensuring they stay clean and tidy. Many people feel that allowing the elderly to pass their days quietly is sufficient.” [Nurse-N2-01]*.

Conversely, family supports significantly motivate nursing home residents to engage in activities. A resident who is highly supported by her son maintains an active lifestyle. She shared that:

*“My son also encourages me to play mahjong. He says it’s a form of exercise*,* so I entertain myself while exercising…If I can’t reach a tile*,* I have to stretch and even stand up*,* moving my hands and feet a bit.” [Resident-N2F-08]*.

Family bonds also serve as a source of inner strength and hope for some residents, motivating them to stay active to achieve their ideal status when with their loved ones. A staff member leveraged family bonds to encourage participation and explained:

*“We guide them with specific scenarios. For example*,* most of our older adults have grandchildren*,* right? Then*,* we might say*,* ‘Look*,* if you don’t be active*,* you won’t be able to take care of your grandchildren. What do you think?’ Hmph*,* it’s a way to coax them into getting some exercise.” [Manager-N2-07]*.

(4) Macrosystem: Societal factors enhance sustainability of activity interventions

Supportive financial policies for the nursing home helped to alleviate cost concerns for both staff members and residents. Financial input, whether from residents or government, is considered essential for implementing physical activity interventions in nursing homes due to the cost associated with such intervention. One staff member expressed:

*“Actually*,* physical activity interventions are costly. If it could be purchased as a service by clients* [residents] *or supported by financial policies*,* we could implement it more effectively.” [Nurse-N2-01]*.

Cost concerns were also a priority for residents, who felt that financial issues should be addressed from the outset if the intervention incurs expenses. Adapting financial policies to accommodate residents’ varying financial situations would make any cost-incurring interventions more accessible. As one resident noted:

*“That* [Cost] *is actually a big issue. This varies from person to person. Although national health insurance might cover part of it…for those with heavy family burdens*,* it’s still difficult for them to manage. It’s important not to take a uniform approach… the cost issue should be addressed right from the beginning.” [Resident-N1M-03]*.

Social funding by the state or donations were used as incentives to reward residents’ participation in physical activities. One staff member explained:

*“From our donations*,* we take a small part of the money* [to give to the resident] *as a reward every time they complete activity tasks.” [Manager-N2-07]*.

Societal ties with the community external to the nursing home boosted engagement in activities. Volunteer visits involving children, students, and community members allowed residents to connect with people outside the nursing home, creating a sense of excitement and social value. As one staff member described:

*“We might have a big event every month where volunteers come to play games and do activities with our residents. They get a chance to connect with people from outside*,* and sometimes even see children*,* which really lifts their spirits and brings them some happiness…sometimes*,* a few of our residents who love singing will even go up on stage during these events and sing on their own.” [Manager-N2-05]*.

## Discussion

### Summary of findings and theory of change refinement

**Fig. 2:**
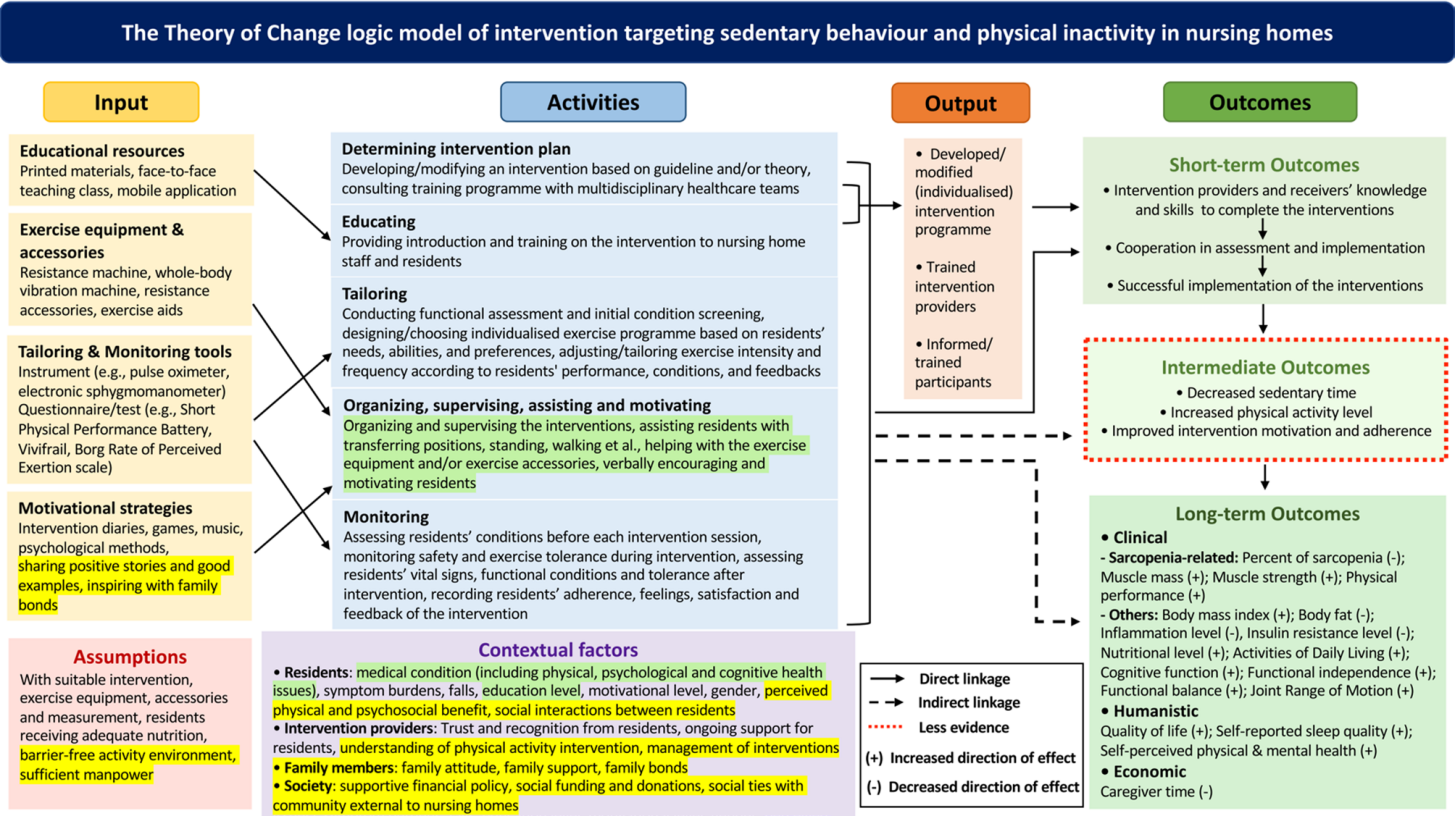
Refined Theory of Change logic model of intervention addressing sedentary behaviour and physical inactivity in nursing homes. Yellow highlight: from qualitative study, Green highlight: from both qualitative study and systematic review. The original Theory of Change logic model was published in “Sarcopenia interventions in long-term care facilities targeting sedentary behaviour and physical inactivity: A systematic review” [32].

The barriers and facilitators to interventions addressing sedentary behaviour and physical inactivity in nursing homes are complex, encompassing four systems: residents’ constraints and perceptions of benefit (microsystem), social interactions, pivotal roles of staff, insufficient activity management and resources constraints within nursing homes (mesosystem), family attitude and involvement (exosystem) and broader societal supports (macrosystem). Building on these findings, a Theory of Change logic model of interventions addressing sedentary behaviour and physical inactivity was refined for the context of nursing homes, integrating results from the qualitative study and preceding systematic review [32] **(**Fig. 2**)**. The refinement emphasises contextual factors, particularly the influence of social interactions between nursing home residents, family members’ roles which were influenced by the culture of filial piety, and broader societal elements (i.e., sufficient funding, societal ties with community external to the nursing homes) within Chinese context. Financial support from societal level, whether through policy initiatives or funding donation, would be helpful. Factors, such as individual constraints and the pivotal role of staff, were corroborated in the qualitative findings.

### Micro-Mesosystem: Enhancing staff roles

Challenges faced by individuals, including physical, psychosocial, cognitive, and educational constraints, heighten the importance of staff roles as organisers, assistance providers, and motivators in physical activity interventions. Individuals with physical limitations often require additional assistance to safely engage in activities, necessitating staff to provide physical support and modify exercises to match their abilities. Similarly, people with cognitive impairments demand extra guidance and supervision, as they may struggle to follow instructions or maintain concentration [37]. Both groups place a demand on staff resources, requiring more personalised attention. This demand can create a conflict for nursing home registered nurses and healthcare assistants, who must balance essential tasks for activities of daily living, such as feeding, personal hygiene, and medication management, with organising physical and recreational activities that contribute to residents’ well-being [38].

The study identified that the tension between meeting timely personal needs and promoting physical activities often leads to rushed work. To save time, staff frequently take over tasks that residents could complete themselves or with some assistance, as allowing residents to do so would take longer. However, they overlook the fact that while residents are performing daily living activities, they are actually also engaging in physical activity and breaking sedentary time. This mirrors findings by a cross-sectional study [39], which noted that nursing home staff frequently handled tasks unnecessarily, despite residents’ capabilities to perform them alone. It is crucial for staff to recognise that encouraging residents to engage in daily activities and fostering their independence can reduce their workload while addressing both care and physical activity needs.

Additionally, psychosocial barriers like negative self-image and embarrassment often emerge when individuals struggle with activities that exceed their physical abilities. Research indicates that non-tailored interventions can lead to discrimination, emotional distress, feelings of exclusion, and reduced engagement [37]. Nursing home staff play a crucial role in delivering tailored physical activity interventions aligned with individual’s functional ability and needs, to ensure adherence and safety [40]. By fostering an inclusive and supportive environment, staff can help mitigate psychosocial burdens while encouraging participation. Despite these challenges, perceived physical improvements and psychosocial benefits, such as increased strength, fulfilment, distraction from homesickness, and dignified life, motivate continued participation. Even modest gains in physical capacity can enhance residents’ confidence and quality of life [41].

### Mesosystem: Refining activity management

Insufficient activity management in nursing homes is the major barrier for physical activity intervention provision. The understanding of these interventions among nursing home staff, which is crucial for intervention implementation, is highly influenced by their education and work experiences [10]. In China, most front-line nursing home workers are women aged 40–59 years, often with low education levels and inadequate training [42]. This demographic tends to have a narrow understanding of physical activity interventions, viewing them as suitable only for residents who are physically and cognitively capable. This perspective aligns with findings that such interventions were often deemed unnecessary for residents with substantial functional decline [43]. A broader definition of physical activity, encompassing various movements and daily tasks, helps mitigate the negative effects of inactivity among nursing home residents.

In this study, current practices tailor activities based on the prolonged staff-resident relationship and understanding of residents’ conditions with their experiences and observations. While this reflects the concept of person-centred care, a strong foundation of knowledge and skills is essential for appropriately tailoring activities for residents [44]. For staff members with limited expertise, this approach may lead to a diluted impact. Incorporating evidence-based practice and systematic assessment may help bridge the gaps in understanding and enhance intervention implementation [32]. Additionally, our finding of “better safe than sorry” mentality indicates that unclear risk accountability leads to overly cautious approaches that restrict residents’ opportunities for physical engagement. Safety laws and regulations can also limit participation in physical activities [10]. Therefore, clear guidelines and risk management protocols are essential for implementing physical activity programmes in nursing homes. Utilising evidence-based practices that adhere to nursing home physical activity guideline [40], along with regular systematic assessment (e.g., Economic, Clinical, and Humanistic Outcomes) [32], can help manage related risks, ensure scientifically sound practice, and provide timely feedback. This approach will optimise the nature and intensity of physical activity interventions, maximising both safety and benefits for residents.

### Mesosystem: Leveraging social interactions strategically

Nursing homes function like small societies, where social interactions significantly influence residents’ engagement in physical activity. Managing ongoing relationships and daily interactions is essential to reduce residents’ stress and burden on others [45]. Socialising during exercise and a sense of collectivity are valued by residents, with group exercise often preferred over individual ones [10]. Peer support during exercise positively contribute to the psychological well-being of nursing home residents, while a spirit of competition can also motivate participation [37]. Residents who model positive behaviour by actively participating in physical activities can empower and inspire others, significantly increasing overall participation and fostering a more active community [46]. Moreover, interactions between staff and residents enable physical activity interventions. Staff members who build strong, trusting relationships with residents and use exceptional motivational skills can greatly enhance residents’ physical activity levels. Identifying these staff members as “champions” can inspire residents and enhance their self-efficacy, fostering a greater sense of capability and motivation to participate in physical activities [9].

This small society-like environment provides a unique opportunity to leverage these interactions to promote intervention targeting sedentary behaviour and physical inactivity. However, being mindful of the potential negative effects of social dynamics is essential. Our findings indicate that peer stress, such as pressure to keep up with others, can undermine residents’ confidence and create feelings of exclusion. Over-competition can further exacerbate these issues and deter engagement [46]. Therefore, the key is to strike a balance that promotes support and motivation while avoiding the pitfalls of peer stress and over-competition. Understanding the social structure within the nursing home and training staff to facilitate positive social interactions and recognise signs of peer stress will help create harmonious and supportive activity groups.

### Exosystem: Maintaining family involvement

Family involvement in nursing homes is crucial for enhancing the well-being of residents, particularly through emotional attachment and shared decision-making shaped by filial piety. In Confucianism, filial piety emphasises respect, care and family continuity, promoting a sense of responsibility that remains integral to Chinese culture, even amidst modernisation [47]. The value of filial piety instils residents with a sense of hope and emotional reliance on their families as they anticipate care and supports from family members [48]. Simultaneously, a sense of duty drives family members to actively engage in their parents’ lives, ensuring they receive adequate care and attention in nursing homes [49]. However, this strong feeling of obligation can sometimes cause tension in shared decision-making. Our findings exhibit family members’ overprotectiveness towards their loved ones, viewing physical activity as unnecessary when basic needs are met. They tend to avoid perceived risks and expect staff to take full responsibility for any incidents, complicating the implementation of physical activity interventions in nursing homes.

Research reported that family involvement through visits and support in daily tasks enhances residents’ well-being and quality of care [50]. Notably, family involvement in physical activities, which often overlooked in existing evidence, may help address residents’ emotional needs and reduce tensions regarding shared decision-making between family members and staff. For example, online interactions, like video updates of residents’ activity participation moments and digital monitoring of residents’ health indicators, keep families stay connected afar. Direct involvement in supporting sedentary time reduction and physical activity sessions [9] fosters both physical and emotional supports and encouragement to residents. This process facilitates residents’ feeling of being supported by their families while enabling families to recognise the intervention benefits, reinforcing their positive attitudes to the interventions. Moreover, it creates a relaxed and engaging communicate environment for families and staff members, helping to reduce tension in shared decision-making. Further studies are warranted to explore effective strategies to integrate family involvement in physical activity interventions within nursing home settings.

### Macrosystem: Sustaining financial input and collaborating with social organisations

Funding factors, including financial policies, resident payments, and donations, influence the provision of physical activity interventions in nursing homes. Cost concerns, such as personnel, equipment, and training expenses, impact decisions on intervention implementation and participation. A one-year study found physical activity interventions could be cost-saving in the long term [51], but sustaining initial investments remains a challenge. Long-term care financial strain is a global issue. In China, out-of-pocket payments dominate long-term care funding, with limited public support. While pilot long-term care insurance has eased burdens in 49 cities since 2016, over 90% still face challenges [52]. This mirrors the situation in Organisation for Economic Co-operation and Development (OECD) countries, where long-term care costs can exceed national median incomes, putting 42–95% of older adults at risk of poverty without public benefits [53]. The global aging population, expected to grow from 12 to 22% by 2050 [54], further strains fiscal capacities for adequate long-term care [53].

Our findings suggest strategies to alleviate financial burden of physical activity interventions, like utilising volunteers, partnering with local schools and hospitals and diversifying funding through donations and charitable foundations. Social ties with community external to nursing homes were identified as an important facilitator to residents’ activity engagement. This supports previous studies indicating that volunteer-led physical activity interventions have positive effect on intervention adherence and health outcomes for older adults, including quality of life, mobility, and general health of nursing home residents [55, 56]. Social voluntary work helps to establish new contacts, which are often viewed as enriching older adults’ lives [56]. Utilising social volunteers and partnering with schools and hospitals not only reduce costs of physical activity interventions, but also strengthen residents’ social ties, fostering a more sustainable and socially enriching environments.

### Strengths and limitations

This study was theoretically informed in its topic guide development and data analysis, grounded in a systematic review of interventions addressing sedentary behaviour and physical inactivity in nursing homes. This design enhances the study’s rigour and ensure that its findings contribute meaningfully to strategies for promoting active behaviours among nursing home residents. Additionally, having separate interviews for managers, nurses and healthcare assistances allowed staff to express their views openly, without being affected by hierarchical dynamics, thus expressing their views more accurately. However, several limitations need to be acknowledged. First, the exclusion of residents lacking capacity to provide informed consent, particularly those with severe cognitive impairment, may restrict the breadth and depth of the findings, as their experiences and perspectives are not captured. Second, the study recruited participants only from urban nursing homes in China, which may limit the generalisability of the findings. This region has unique cultural and socio-economic characteristics, including a strong sense of family-oriented care, distinct healthcare infrastructure, and specific local attitudes towards aging and physical activity. While this context enhances the relevance of the findings locally, it may constrain their applicability to other countries or regions with different cultural, social, or healthcare settings. Additionally, the unique characteristics of urban facilities, such as access to resources and staffing patterns, may differ from those in rural areas, potentially affecting the transferability of the results to a broader population of nursing homes.

### Contribution to the literature

This study makes three key contributions to the existing literature. First.it addresses an important empirical gap by exploring sedentary behaviour and physical inactivity in the under-researched context of urban Chinese nursing homes. Most existing studies in this field have been conducted in Western settings, and a few have included perspectives from Chinese care institutions. Second, the study contributes methodologically and theoretically by refining an existing ToC logic model on intervention addressing sedentary behaviour and physical inactivity [32] based on context-specific qualitative findings. This refinement enhances the relevance and applicability of this theoretical framework in Chinese nursing homes and demonstrates how qualitative research can support the local adaptation of theoretical frameworks. Third, the study findings revealed that the sedentary behaviour and physical inactivity should be understood as system issues. Using the lens of the ecological social theory, the findings provide contextual insights into how the intervention should be adapted to address the multilevel factors that may influence its implementation in nursing homes.

### Recommendation for further research

Future research should explore the impact of family involvement in physical activity interventions for nursing home residents, particularly its effects on motivation, adherence, and long-term functional outcomes. Studies should also examine how organisational factors, such as staffing capacity, resource availability, and organisational culture, shape the feasibility and sustainability of these interventions. Additionally, cross-cultural research is needed to understand how social norms, attitudes toward ageing, and caregiving practices influence intervention implementation in different healthcare systems. Such insights would support the development of culturally responsive and scalable strategies, ultimately enhancing engagement and improving older adults’ quality of life globally.

### Implication for policy and practice

It is recommended that consistent, evidence-based training for nursing home staff be prioritised to enhance their understanding and skills in delivering physical activity interventions. This approach could improve the quality and effectiveness of such interventions. Policymakers may also wish to consider defining acceptable risks associated with physical activity, ensuring safety regulations are in place without restricting residents’ opportunities for engagement. Nursing home staff are encouraged to identify social interactions within nursing home settings and, where appropriate, leverage these interactions to facilitate the implementation of physical activity interventions. Additionally, maintaining family involvement, whether through online or offline communication and participation, could help ensure more effective practice. Sustained government investment and policy frameworks that encourage collaboration between social organisations and nursing homes could help integrate physical activity into routine care, ultimately improving quality of life and functional independence among older adults.

## Conclusion

This study identified multilevel factors from the individual, organisational, family, and societal contexts that influence interventions addressing sedentary behaviour and physical inactivity in nursing homes. Multilevel strategies from enhancing staff roles, refining activity management, leveraging social interactions within nursing homes strategically, maintaining family involvement, sustaining financial input and collaborating with social organisations are crucial to address these challenges. The adaptation of the Theory of Change logic model to specific context opens opportunities for international collaboration in tailoring and applying the model in diverse cultural settings.

## Supplementary Information

Supplementary Material 1.

Supplementary Material 2.

## Data Availability

The data supporting the findings of this study consist of qualitative interviews with nursing home residents and staff, which contain personal and sensitive information. Due to privacy and ethical concerns, these data cannot be made publicly available. However, anonymised excerpts from the interviews may be available from the corresponding author upon reasonable request and subject to approval by the relevant ethics committee. Access to the data will require a formal data-sharing agreement to ensure the confidentiality of participants is maintained.
