# Correction: Barriers and facilitators to addressing sedentary behaviour and physical inactivity among nursing home residents: a qualitative study

**DOI:** 10.1186/s12877-025-06578-1

**Published:** 2025-11-29

**Authors:** Yihan Mo, Helen Yue-lai Chan, Yuxin Zhou, Linghui Chen, Guanxiu Tang, Anna Bone, Matthew Maddocks, Catherine Evans

**Affiliations:** 1 Cicely Saunders Institute of Palliative Care, Policy and Rehabilitation, King’s College London, London, UK; 2 The Nethersole School of Nursing, Faculty of Medicine, The Chinese University of Hong Kong, Hong Kong, SAR, China; 3 Department of Nursing, Zhongda Hospital Southeast University, Nanjing, China; 4 Department of Geriatrics, The Third Xiangya Hospital, Central South University, Changsha, China

**Correction: BMC Geriatr 25**,** 648 (2025)**

10.1186/s12877-025-06272-2

Following publication of the original article [1], the authors identified an error in the author name of Helen Yue-lai Chan.

The incorrect author name is: Helen Chen.

The correct author name is: Helen Yue-lai Chan.

The author group has been updated above and the original article [1] has been corrected.
