# Supplementary material for: Barriers and facilitators to addressing sedentary behaviour and physical inactivity among nursing home residents: a qualitative study
Source: BMC Geriatr. 2025 Aug 20;25:648. doi: 10.1186/s12877-025-06272-2 (PMC12366238; doi:10.1186/s12877-025-06272-2)
Supplement: Supplementary file 1 — Supplementary Material 1. [file 12877_2025_6272_MOESM1_ESM.docx]

**Individual interview topic guide – For residents**

Open questions on personal context: Can you please tell me a little about yourself? (Prompts: -How long have you lived in nursing home, -what was the condition when you moving to here, -why are you interested in coming today?)

| **Question area** | **Prompts** |
| --- | --- |
| **Topic area 1: Current health, function, and concerns** | |
| 1. How are you feeling generally living in the nursing home? | -How do you feel about your general health and mood living here?  -How do you think of services provided in the nursing home?  -How do you think of the environment of the nursing home?  -How do you like the people around you? (e.g., other residents, nurses) |
| 1. What sorts of things are important to you living in the nursing home? | -E.g., living independently, maintaining physical performance, relieving symptom burdens, controlling health issues, keeping in good mood, spending time with friends / joining social activities |
| 1. Can you please describe what things you do in the nursing home from when you wake to going to sleep at night? | - E.g., sitting / lying / reclining activities, physical activities, social activities |
| **Topic area 2: Experience in sedentary time reduction and physical exercise (barriers & facilitator)** | |
| 1. Can you please describe any experience in reduce sitting/lying/reclining time and/or physical exercise in daily life? | **If have the related experience**  - How do you reduce your sedentary time? What kind of physical exercise you usually perform (e.g., running, Taiji, Qigong, Ping-pong tennis, resistance exercise)?  - What is the frequency and intensity?  - What encourages you to reduce sedentary time / do [name(s) of physical activity(ies)]/ in daily life? (e.g., good for health (physical and/or psychological), social influence (friends, family, staff), sense of supervised, reward)  - What makes it difficult for you to reduce sedentary time / do [name(s) of physical activity(ies)] in daily life? (e.g., diseases, symptom burdens, physical limitations, environmental/resources limitations, lack of skills or any supports). Have you addressed them? If yes, how? If no, do you have any plan to address them, do you have confidence to overcome them?  **If don’t have the related experience**  **- Go to question 5-6** |
| 1. What may prevent you from reduce sitting/lying/reclining time and doing physical exercise in daily life? | -E.g., subjectively unwillingness, diseases, symptom burdens, physical limitations, environmental/resources limitations, lack of skills or any supports |
| 1. What will make you reduce sitting/lying/reclining time and undertake physical exercise in daily life? | -E.g., supports for any limitations (physical, environmental etc.), social influences, supervisions, reward, guidance from skilled staff |
| **Additional questions** | |
| 1. Thank you, your answers are helpful. Before finish, is there anything you would like to talk about? | -How do you feel about having this conversation? |

**Interview topic guide - For healthcare assistants**

Open questions on personal context: Can you please tell me a little about yourself? (Prompts: -Background, -how long have you been working in nursing home, -why are you interested in coming today?)

| **Questions area** | **Prompts** | |
| --- | --- | --- |
| **Topic area 1: Current working content and experience of delivering exercise-focused interventions** | | |
| 1. Can you please describe what things you do in a working day in nursing home from pre-work preparation to getting off work? |  | |
| 2. What components of your work are related to reducing sitting/lying/reclining time and improving physical activity for nursing home residents? | -E.g., guiding regular exercise sessions, supporting them to walk from their home to dining hall, reminding them to get up or stand up frequently | |
| 3. How do you think of the part of work related to reducing sitting/lying/reclining time and improving physical activity for nursing home residents? | -How could this part of work benefit nursing home residents?  -What can be done on this part of work to meet every resident’s needs and satisfaction? | |
| 4. Are there any factors that might influence you to facilitate breaks in sitting/lying/reclining time and physical activity among adults living in nursing home? | -E.g., lack of suitable environment/equipment/space/useful monitoring system, time constraints, heavy nursing work | |
| **Additional questions** | | |
| 5. Thank you, your answers are helpful. Before finish, is there anything you would like to talk about? | | -How do you feel about having this conversation? |

**Interview Topic guide --- For senior staff (managers, registered nurses)**

Open questions on personal context: Can you please tell me a little about yourself? (Prompts: -Background, -how long have you been working in nursing home, -why are you interested in coming today?)

| **Questions area** | **Prompts** | |
| --- | --- | --- |
| **Topic area 1: Current working content and experience of delivering exercise-focused interventions** | | |
| 1. Can you please tell me about the various services provided by your nursing home for the residents? |  | |
| 2. What components of these services (your work content) are related to reducing sitting/lying/reclining time and improving physical activity for nursing home residents? | -E.g., guiding regular exercise sessions, supporting them to walk from their home to dining hall, reminding them to get up or stand up frequently | |
| 3. What do you think of the part of services (your work content) related to reducing sitting/lying/reclining time and improving physical activity for nursing home residents? | -How could this part of work benefit nursing home residents?  -What can be done on this part of work to meet every resident’s needs and satisfaction? | |
| 4. Are there any factors that might influence the quality of the services (your work content) related to reducing sitting/lying/reclining time and improving physical activity for nursing home residents? | -E.g., lack of suitable environment/equipment/space/useful monitoring system, financial concerns, time constraints, heavy nursing work | |
| **Additional questions** | | |
| 5. Thank you, your answers are helpful. Before finish, is there anything you would like to talk about? | | -How do you feel about having this conversation? |
